# Antioxidant Enzymatic Activity and Osmotic Adjustment as Components of the Drought Tolerance Mechanism in *Carex duriuscula*

**DOI:** 10.3390/plants10030436

**Published:** 2021-02-25

**Authors:** Peichen Hou, Feifei Wang, Bin Luo, Aixue Li, Cheng Wang, Lana Shabala, Hassan Ahmed Ibraheem Ahmed, Shurong Deng, Huilong Zhang, Peng Song, Yuhong Zhang, Sergey Shabala, Liping Chen

**Affiliations:** 1 Beijing Research Center of Intelligent Equipment for Agriculture, Beijing Academy of Agriculture and Forestry Sciences, Beijing 100097, China; houpc@nercita.org.cn (P.H.); luob@nercita.org.cn (B.L.); liax@nercita.org.cn (A.L.); wangc@nercita.org.cn (C.W.); 2 Tasmanian Institute of Agriculture, University of Tasmania, Tasmania 7001, Australia; L.Shabala@utas.edu.au (L.S.); hassan.ahmed@utas.edu.au (H.A.I.A.); 3 Jiangsu Key Laboratory of Crop Genetics and Physiology/Co-Innovation Center for Modern Production Technology of Grain Crops, Jiangsu Key Laboratory of Crop Genomics and Molecular Breeding/Key Laboratory of Plant Functional Genomics of the Ministry of Education, College of Agriculture, Yangzhou University, Yangzhou 225009, China; Feifei.Wang@yzu.edu.cn; 4 International Research Centre for Environmental Membrane Biology, Foshan University, Foshan 528011, China; 5 Department of Botany, Faculty of Science, Port Said University, Port Said 42526, Egypt; 6 State Key Laboratory of Tree Genetics and Breeding, The Research Institute of Forestry, Chinese Academy of Forestry, Beijing 100091, China; dengsr@caf.ac.cn (S.D.); zhangyuhong512008@163.com (Y.Z.); 7 Tianjin Research Institute of Forestry of Chinese Academy of Forestry, Tianjin 300000, China; hlzhang@caf.ac.cn; 8 College of Plant Science and Technology, Huazhong Agricultural University, Wuhan 430070, China; songp@mail.hzau.edu.cn

**Keywords:** *Carex duriuscula*, drought tolerance, ROS, antioxidant enzyme, ion concentration, osmotic adjustment, energy cost

## Abstract

Drought stress is a major environmental constraint for plant growth. Climate-change-driven increases in ambient temperatures resulted in reduced or unevenly distributed rainfalls, leading to increased soil drought. *Carex duriuscula* C. A. Mey is a typical drought-tolerant sedge, but few reports have examined the mechanisms conferring its tolerant traits. In the present study, the drought responses of *C. duriuscula* were assessed by quantifying activity of antioxidant enzymes in its leaf and root tissues and evaluating the relative contribution of organic and inorganic osmolyte in plant osmotic adjustment, linking it with the patterns of the ion acquisition by roots. Two levels of stress—mild (MD) and severe (SD) drought treatments—were used, followed by re-watering. Drought stress caused reduction in a relative water content and chlorophyll content of leaves; this was accompanied by an increase in the hydrogen peroxide (H_2_O_2_) and superoxide (O_2^−^_) contents in leaves and roots. Under MD stress, the activities of catalase (CAT), peroxidase (POD), and glutathione peroxidase (GPX) increased in leaves, whereas, in roots, only CAT and POD activities increased. SD stress led to an increase in the activities of CAT, POD, superoxide dismutase (SOD), and GPX in both tissues. The levels of proline, soluble sugars, and soluble proteins in the leaves also increased. Under both MD and SD stress conditions, *C. duriuscula* increased K^+^, Na^+^, and Cl^−^ uptake by plant roots, which resulted in an increased K^+^, Na^+^, and Cl^−^ concentrations in leaves and roots. This reliance on inorganic osmolytes enables a cost-efficient osmotic adjustment in *C. duriuscula*. Overall, this study revealed that *C. duriuscula* was able to survive arid environments due to an efficient operation of its ROS-scavenging systems and osmotic adjustment mechanisms.

## 1. Introduction

Drought is one of the most important abiotic stress factors affecting plant growth in arid and semi-arid regions worldwide [1,2]. Drought is also an important social factor [3], affecting the livelihood of billions of people. About 45% of agricultural land worldwide suffers from frequent or persistent drought stress [4]. Drought stress affects plant growth and development (and, ultimately, yield) by reducing water availability, thus affecting photosynthesis and respiration; it also causes loss of the cell turgor and alters source–sink relationships and metabolic balance [1,5]. Drought stress also leads to increased production of reactive oxygen species (ROS), such as superoxide (O_2^−^_), hydrogen peroxide (H_2_O_2_), singlet oxygen (^1^O_2_), and hydroxyl radical (OH∙) [6]. ROS plays a dual-edged sword role, acting as signaling molecules in abiotic stress signaling at low concentrations [7,8] and causing damage to key macromolecules when present in excessive quantities. In their signaling role, ROS regulate cytosolic ion homeostasis by regulating the activity of various ROS-sensitive ion channels [9,10]. At the same time, oxidation by ROS destroys plant cell membranes and chloroplasts and induces membrane lipid peroxidation, producing the toxic metabolite malondialdehyde (MDA). The production of ROS also damages proteins, lipids, and nucleic acids, causing cell death [11,12,13].

To withstand the damage from the high content of ROS that results from the drought, plants have evolved enzymatic and non-enzymatic ROS scavenging systems, to maintain the optimal ROS balance required for cell metabolism. Detoxification by antioxidant enzymes is arguable the most effective way to remove ROS in plants; the key enzymes include superoxide dismutase (SOD), catalase (CAT), peroxidase (POD), ascorbate peroxidase (APX), glutathione peroxidase (GPX), and glutathione reductase (GR), monodehydroascorbate reductase (MDHAR), dehydroascorbate reductase (DHAR), guaicol peroxidase (GOPX), and glutathione-S- transferase (GST) [13,14]. SOD is localized in chloroplasts, mitochondria, peroxisomes and cytoplasm [15]. SOD is the first line of defense against ROS-induced damage in plant antioxidant systems, converting superoxide radical into H_2_O_2_ [16]. CAT is localized in a peroxisome [17], where it can remove H_2_O_2_, a product of the fatty acid beta-oxidation, photorespiration, and purine metabolism, by converting it to oxygen and water via a catalytic reaction 2H_2_O_2_ → O_2_ + 2H_2_O [18]. POD is localized in organelles such as chloroplasts, mitochondria and vacuoles [19,20,21], assisting in the removal of the H_2_O_2_ produced by the SOD disproportionation reaction [15,22]. Both APX and GPX have an H_2_O_2_ scavenging role [13] and are localized in chloroplasts, mitochondria, and cytoplasm [23,24]. APX is considered as a key enzyme for the removal of H_2_O_2_ in chloroplasts, although its response to drought stress varies among species [13]. The non-enzymatic systems mainly include glutathione, ascorbic acid (AsA), flavonoids, carotenoids, tocopherols, and phenolics and other low-molecular-weight antioxidants, which mediate the complete removal of singlet oxygen (^1^O_2_) and hydroxyl radicals (OH∙) [25,26,27].

Reduced water availability causes an osmotic stress that interferes with cell metabolism and affects plant growth and development. To alleviate the osmotic balance damage caused by drought stress, plants synthesize and accumulate compatible solutes (CS) such as soluble sugars (SS), polyols, amino acids and quaternary amines [28,29,30,31,32]. An alternative option is to accumulate the inorganic ions (potassium, sodium, chloride); this option allows plants to reduce water potential in a more cost-effective way and thus resist osmotic stress [29,32,33,34,35,36]. K^+^ is the main cytosolically abundant cation that is ideally suitable for osmotic adjustment purposes without causing cellular toxicity. Na^+^ and Cl^−^ are also rapidly absorbed and accumulated when plants are subjected to osmotic stress and participate in the osmotic adjustment of plants as the main inorganic osmotic regulators [31,37]. Plants also stabilize the structure of cells and proteins by accumulating soluble proteins (SP), to resist drought stress [15].

*Carex duriuscula* C. A. Mey, is a perennial sedge widely distributed in arid and semi-arid regions of northern China [38] that also occurs in Mongolia [39], Russia [40], Canada [41], and North America [42,43]. This species is particularly resistant to drought stress; it has dense roots, tillers strongly, grows rapidly, and tolerates soil nutrient deficiency and repeated harvesting, thus providing abundant wild forage in the vast northern parts of China. Despite its wide practical use, the mechanisms of drought tolerance in *C. duriuscula* are understood very poorly. This gap in the knowledge was addressed in this paper. The finding of this work may be then extrapolated to other species, helping to preserve water resources by breeding drought-resistant and affordable grass species with low water requirements, thereby contributing to land use efficiency in arid and semi-arid areas.

## 2. Results

### 2.1. Phenotypic Effects of Drought Stress

**Figure 1:**
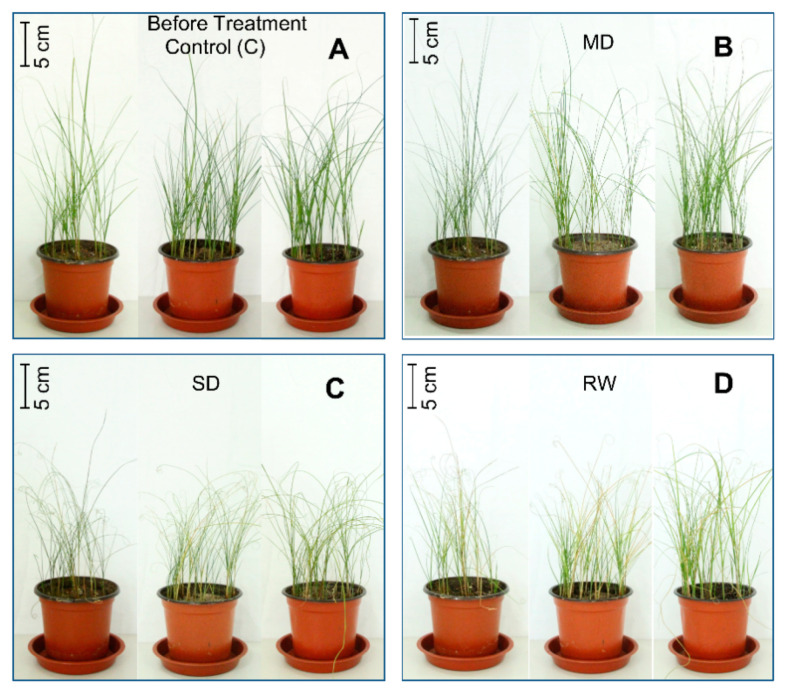
Phenotype of *C. duriuscula* before and after 14 d of drought treatment and after re-watering. (**A**) Control (C): the soil field capacity (FC) was maintained at 75–80%. (**B**) Mild drought (MD), with FC = 40–45%. (**C**) Severe drought (SD) with FC = 20–25%. (**D**) Re-watering (RW); FC = 75–80%.

**Figure 2:**
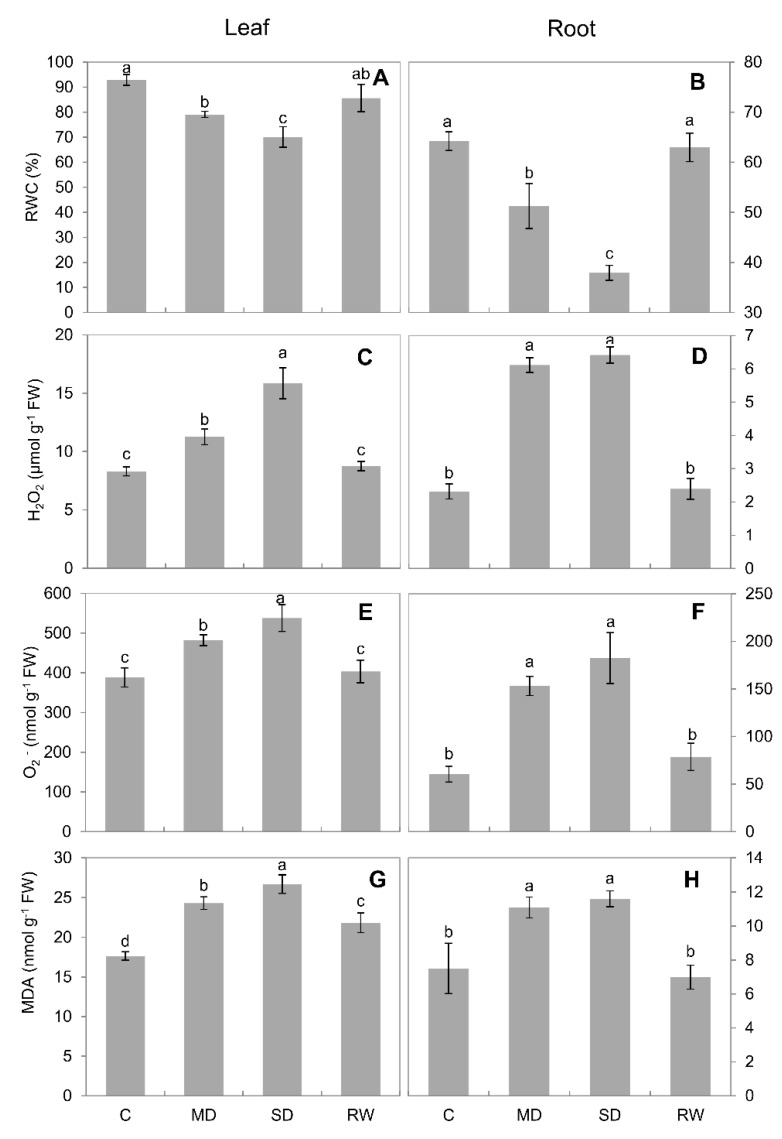
Effects of 14d of drought stress on physiological indicators in leaves and roots of *C. duriuscula*. C-control, MD-mild stress, SD-severe stress, RW-re-watering. (**A**,**B**) relative water content (RWC). (**C**,**D**) H_2_O_2_ content. (**E**,**F**) O_2^−^_ content. (**G**,**H**) MDA content. Data are means ± SE (*n* = 3–6). Data labeled with different lower-case letters are significantly different at *p* < 0.05.

*C. duriuscula* showed typical drought tolerance characteristics. Phenotypic changes were not obvious after MD stress. After SD stress, plants wilted, their leaves yellowed and the tops curled, and plants stopped growing. After re-watering, the base of the plants began to turn green again and their growth began to recover (Figure 1). With increasing drought severity, relative water content decreased, and it was lowest under SD stress (Figure 2A), although the plants survived SD stress (Figure 1). After re-watering, plants resumed normal growth (Figure 1 and Figure 2A,B)

### 2.2. Reactive Oxygen Species and MDA Content

The content of H_2_O_2_ in the leaves and roots under MD and SD stress was significantly (*p* < 0.05) higher than that in the control. Under SD stress, the H_2_O_2_ content in the leaves and roots reached 17.0 ± 2.1 and 7.0 ± 0.7 μmol g^−1^ fresh weight (FW), respectively (Figure 2C,D), indicating more severe oxidative stress to the former tissue. O_2^−^_ content was also increased. Under SD stress, the O_2^−^_ content reached 537 ± 34 and 182 ± 27 nmol g^−1^ FW in the leaves and roots, respectively. After re-watering, it recovered to near the level detected in the control (Figure 2E,F).

Under MD and SD stresses, MDA content in leaves and roots was significantly (*p* < 0.05) higher than in the control, and it was highest under SD stress. After re-watering, MDA content in the leaves and roots declined to approximately the level detected in the control (Figure 2G,H), matching reported ROS production (Figure 2C–F).

### 2.3. Chlorophyll Content

**Figure 3:**
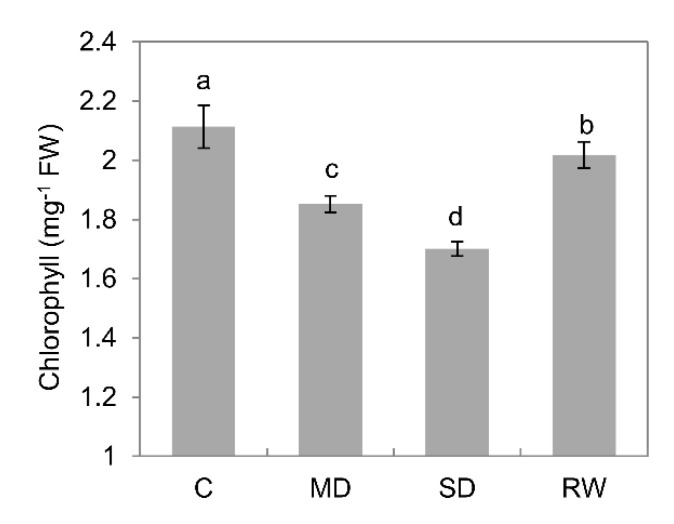
Effects of drought stress on the chlorophyll content of *C. duriuscula*. C-control, MD-mild stress, SD-severe stress, RW-re-watering. Data are means ± SE (*n* = 3-6). Data labeled with different lower-case letters are significantly different at *p* < 0.05.

Drought stress significantly affected chlorophyll content in leaves. Under MD and SD stress, the relative chlorophyll content was reduced by 12.4% and 19.5%, respectively, as compared to controls (Figure 3). Chlorophyll content returned to near the level detected in the control following re-watering (Figure 3).

### 2.4. Activities of Antioxidant Enzymes

**Figure 4:**
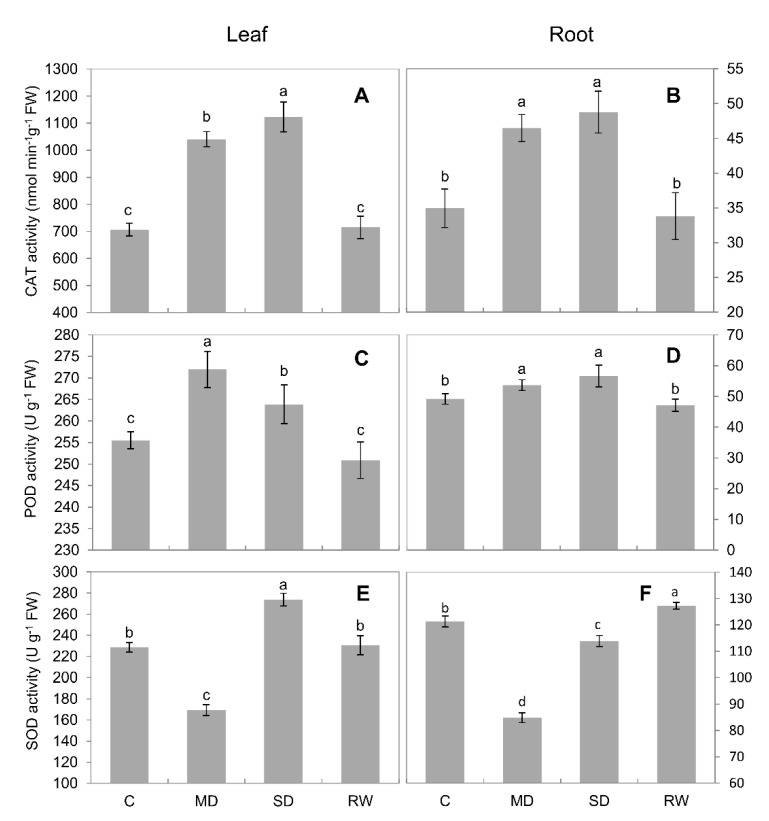
Antioxidant enzyme activity in *C. duriuscula* in response to drought stress. C-control, MD-mild stress, SD-severe stress, RW-re-watering. (**A**,**B**) CAT activity. (**C**,**D**) POD activity. (**E**,**F**) SOD activity. Data are means ± SE (*n* = 3–6). Data labeled with different lower-case letters are significantly different at *p* < 0.05.

**Figure 5:**
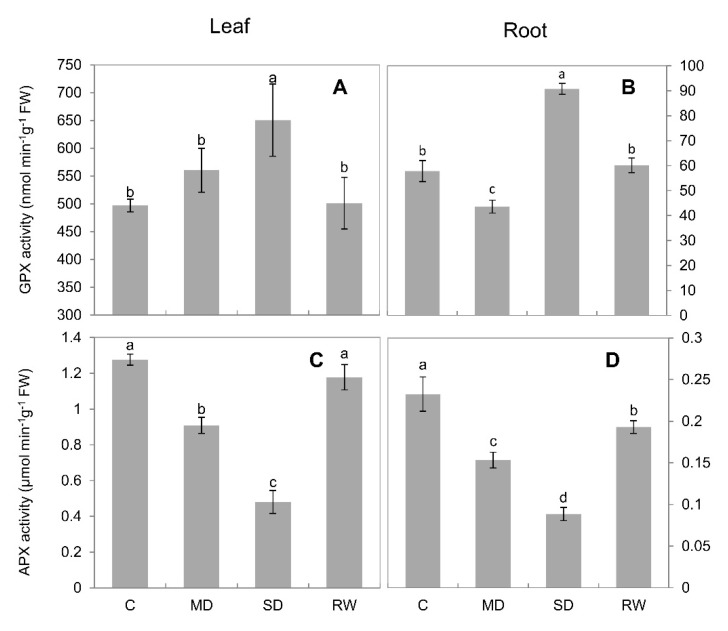
Antioxidant enzyme activity in *C. duriuscula* in response to drought stress. C-control, MD-mild stress, SD-severe stress, RW-re-watering. (**A**,**B**) GPX activity. (**C**,**D**) APX activity. Data are means ± SE (*n* = 3–6). Data labeled with different lower-case letters are significantly different at *p* < 0.05.

CAT activity increased steadily under drought stress. Under MD stress, CAT activity in leaves and roots was increased 47.3% and 32.9%, respectively, as compared to control; this was 58.9% and 39.5% under SD stress. Under both MD and SD stress conditions, CAT activity in the leaves significantly exceeded that in the roots (Figure 4A,B). Drought stress led POD activity to increase significantly (*p* < 0.05), and it decreased upon re-watering (Figure 4C,D). In the leaves, POD activity increased more under MD stress than under SD stress, although it was significantly (*p* < 0.05) higher under both stress treatments than in the control (Figure 4C). The activity of SOD in leaves and roots decreased significantly (*p* < 0.05) under MD stress. However, it increased significantly (*p* < 0.05) under SD stress, compared with the control (an increase of 19.8% in the leaves). After re-watering, SOD activity in the leaves and roots returned to a level similar to that observed in the control (Figure 4E,F). Under MD and SD stresses, GPX activity in the leaves increased significantly (*p* < 0.05); however, it returned to control activity after re-watering (Figure 5A). In contrast, in the roots, MD stress caused GPX activity to decrease, whereas SD stress caused it to increase sharply (Figure 5B). Drought stress caused APX activity to decrease continuously in the leaves and roots, but it increased after re-watering (Figure 5C,D).

### 2.5. Compatible Solutes and Soluble Proteins Content

**Figure 6:**
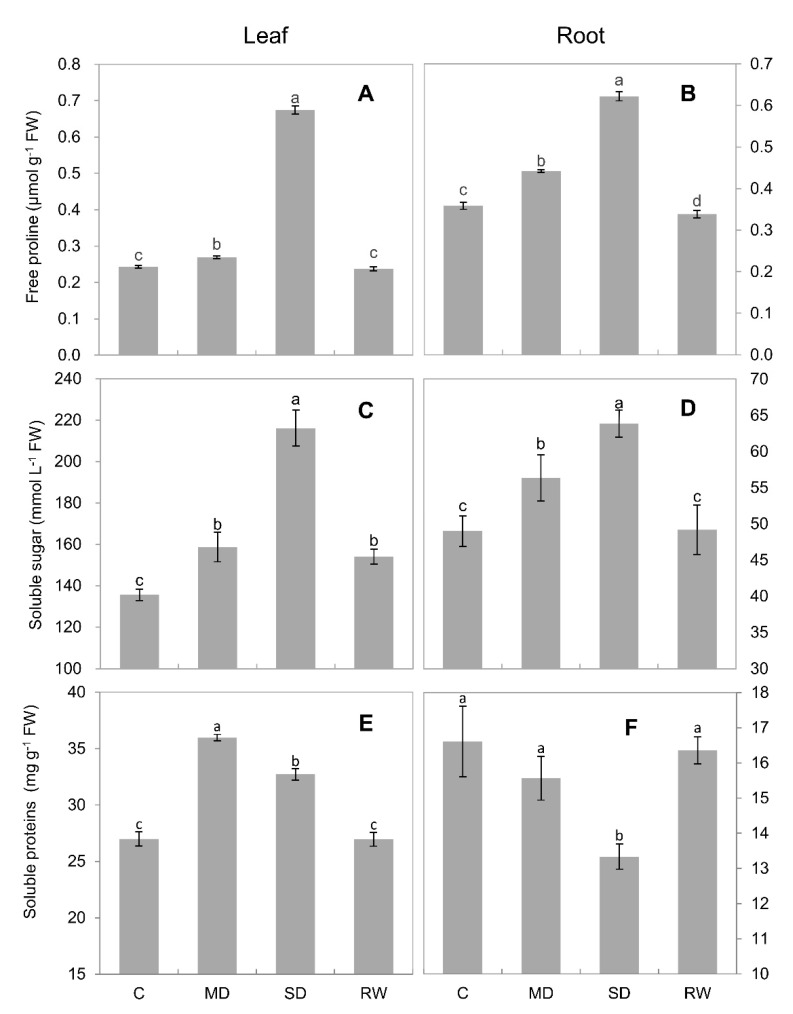
Effects of drought stress on the content of compatible solutes and soluble proteins in *C. duriuscula*. C-control, MD-mild stress, SD-severe stress, RW-re-watering. (**A**,**B**) proline content. (**C**,**D**) soluble sugars content. (**E**,**F**) soluble proteins content. Data are means ± SE (*n* = 3–6). data labeled with different lower-case letters are significantly different at *p* < 0.05.

In the leaves, proline content was not changed under MD stress, whereas under SD stress it increased 1.8 times compared with the control (Figure 6A). In the roots, proline content increased significantly (*p* < 0.05) under both MD and SD stress, by 23.3% and 73.6%, respectively (Figure 6B). After re-watering, proline content in leaves and roots returned to the content observed in the control (Figure 6A,B).

Drought stress caused soluble sugar content in the leaves and roots to increase significantly (*p* < 0.05). For all treatments, the soluble sugar content in leaves was significantly (*p* < 0.05) higher than in roots (Figure 6C,D).

In the leaves, both MD and SD stress caused a significant increase in soluble proteins content, which decreased to the content observed in the control after re-watering (Figure 6E). However, in the roots, soluble protein content decreased relative to that observed in the control, and the decrease was higher under SD than under MD stress; it recovered after re-watering (Figure 6F).

### 2.6. Ion Concentration and Flux

**Figure 7:**
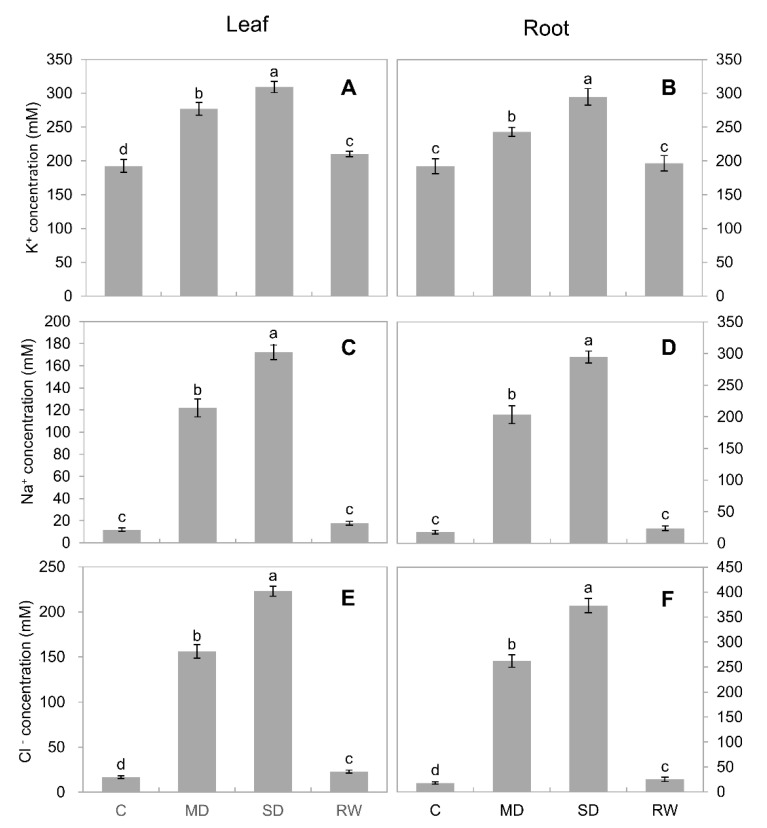
The accumulation of inorganic ions in the leaves and roots of *C. duriuscula* under drought stress. C-control, MD-mild stress, SD-severe stress, RW-re-watering. (**A**,**B**) K^+^ concentration, (**C**,**D**) Na^+^ concentration, (**E**,**F**) Cl^−^ concentration. Data are means ± SE (*n* = 3–6). Data labeled with different lower-case letters are significantly different at *p* < 0.05.

Under drought stress, the concentration of inorganic ions in leaves and roots increased significantly (*p* < 0.05), in a dose-dependent manner (Figure 7). Under control conditions, K^+^ was the major inorganic osmolyte (~200 mM in both leaf and root sap; Figure 7A,B), with Na^+^ and Cl^−^ concentrations in the low millimolar range. Under MD stress conditions, however, the concentration of Na^+^ increased ~10-fold compared with the control (Figure 7C,D); this increase for Cl^−^ was 8- to 15-fold (Figure 7E,F). Under SD stress, the increase was 13- and 15-fold for Na^+^ (Figure 7C,D), and 12- and 21-fold for Cl- (Figure 7E,F) for leaves and roots, respectively, with the concentration of both ions becoming comparable with those for K^+^. At the same time, increases in K^+^ concentration were rather modest (about 50% for SD; Figure 7A,B). After re-watering, the above-mentioned inorganic ions concentration decreased significantly (Figure 7A–F).

**Figure 8:**
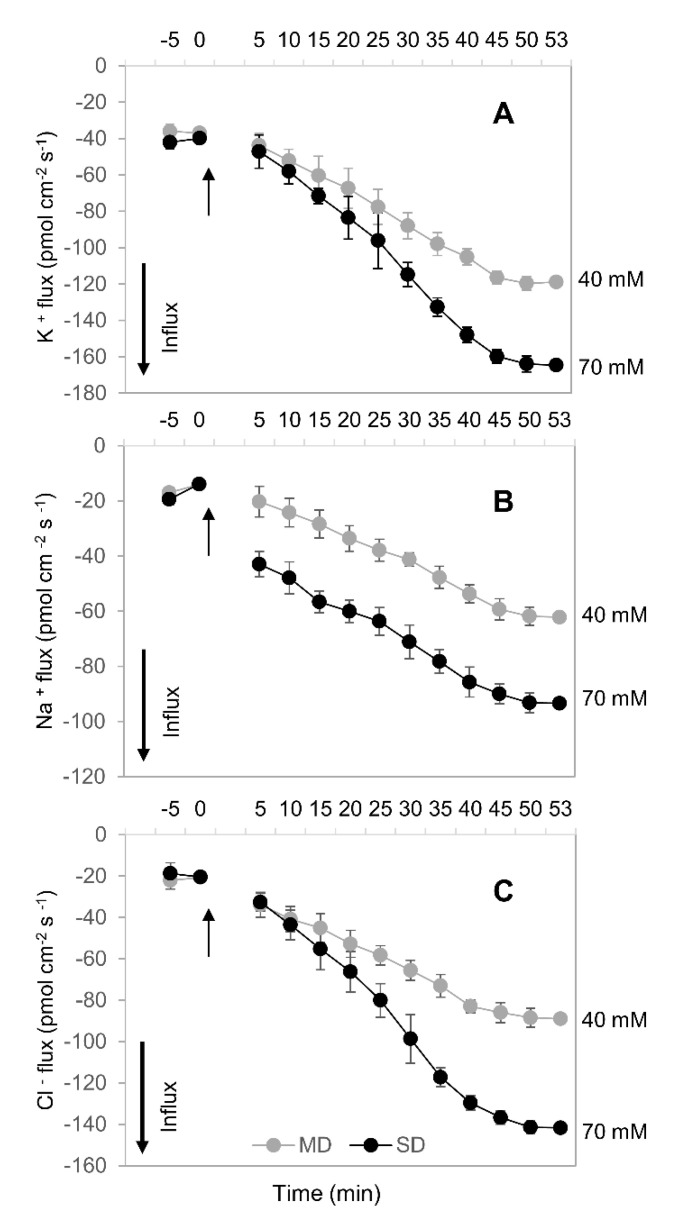
Transient kinetics of net ion fluxes measured from the meristematic region of *C. duriuscula* roots in response to mild (MD; 40 mM mannitol) and SD (70 mM mannitol) osmotic stress. (**A**) K^+^ flux, (**B**) Na^+^ flux, (**C**) Cl^−^ flux. Data are means ± SE (*n* = 3-6). Data labeled with different lower-case letters are significantly different at *p* < 0.05.

In order to explain the mechanistic basis of above drought-stress-induced changes in the tissue ion concentration, we have used ion-selective Na^+^, K^+^, and Cl^−^ microelectrodes to quantify the kinetics of root ion uptake upon hyperosmotic stress. The MD and SD conditions were mimicked by adding appropriate amounts of mannitol to the bath (final concentration 40 and 70 mM, respectively). As shown in Figure 8, mannitol treatment has resulted in rapid (within minutes) increased uptake of all measured ions, reaching a plateau within 40–45 min. This increase was dose-dependent (e.g., higher stimulation by higher mannitol concentrations), matching the ion concentration data reported in Figure 7.

### 2.7. Drought-Stress-Induced Changes in the Sap Osmolarity

**Figure 9:**
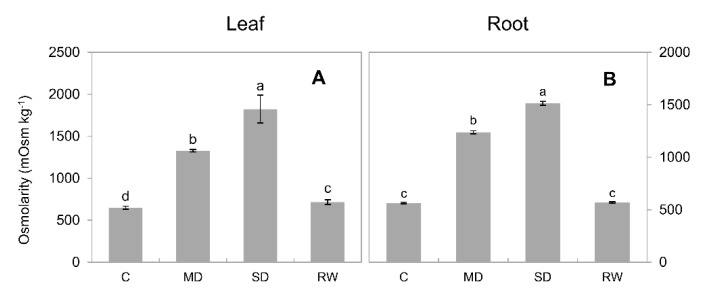
The osmolarity of sap in the leaves and roots of *C. duriuscula* under drought stress. C-control, MD-mild stress, SD-severe stress, RW-re-watering. Data are means ± SE (*n* = 3–6). Data labeled with different lower-case letters are significantly different at *p* < 0.05. (**A**) The osmolarity of leaves and roots in control was 650 ± 18 mOsmol kg−1 under drought stress. (**B**) The osmolarity of leaves and roots in control was 564 ± 6.3 mOsmol kg−1 under drought stress.

The osmolarity of leaves and roots in control was 650 ± 18 and 564 ± 6.3 mOsmol kg^−1^, respectively (Figure 9A,B) and increased significantly (*p* < 0.05) under drought stress. For MD stress, leaf and root osmolarities were 1327 ± 15.0 and 1238 ± 15.0 mOsmol kg^−1^, respectively (*p* < 0.05), and for SD stress, these values were 1823.0 ± 58.0 mOsmol kg^−1^ and 1513 ± 19 mOsmol kg^−1^.

### 2.8. The Relative Contribution of Compatible Solutes and Inorganic Osmolytes towards Osmotic Adjustment

Under control conditions, organic osmolytes were responsible for about 2/3 of the overall osmotic potential in plant tissues (Table 1). The drought stress resulted in a massive (3-fold in leaves and roots, respectively) increase in tissue osmolarity, following the need for osmotic adjustment. While the content of both organic and inorganic osmolytes increased, the major driving force was an increase in inorganic ion uptake. Taking MD stress as an example, the contribution of compatible solutes in leaves decreased from 66% to 58%, and in roots this decrease was even stronger (from 60% to 43%). These changes were a result of the heavy reliance of plants on uptake of Na^+^ and Cl^−^, whose contribution in roots increased from merely 3% under control conditions to 16% and 21% under MD conditions (Table 1). At the same time, the relative contribution of both proline and soluble sugars decreased by about 0.5-fold, respectively (Table 1). Similar trends were also observed for SD treatment. The relative contribution of K^+^ dropped from 34% to 19% in roots and from 30% to 16% in leaves, reflecting its reduced availability under stress conditions.

## 3. Discussion

### 3.1. Drought Stress Limits the Growth of C. duriuscula and Triggers Increase in ROS Content

Understanding the mechanistic basis of plant adaptation to drought environment is instrumental in developing of drought-tolerant genotypes. The present study aimed to investigate the mechanisms of drought tolerance in *C. duriuscula*. We found that, although drought stress affected *C. duriuscula* growth and physiological attributes, it was able to quickly recover when subsequently rewatered. Similarly, although drought stress lowered the relative water content, and induced an increase in ROS content and reduction in chlorophyll content, *C. duriuscula* plants recovered from these effects without major detrimental consequences. This indicated the presence of an effective drought tolerance mechanism(s) in this species.

Hyperosmotic conditions result in a massive increase in ROS content, reported in many species—e.g., sunflower and sorghum [44], tepary bean [45], maize [46], barley [47], peach [48]. This was also the case for *C. duriuscula* here (Figure 2). This increase is predominantly a result of the imbalance between the amount of absorbed light and plant capacity for CO_2_ assimilation [49]. Another major source of ROS under hyperosmotic conditions is the NADPH oxidase (RBOH) [50,51]. Elevated content of ROS aggravates membrane lipid peroxidation and causes cells to produce large amounts of MDA [52]. We observed an increase in ROS in *C. duriuscula* tissues under drought stress, which changed the oxidative balance in the cell, causing the plant to undergo membrane lipid peroxidation, and further explained the large amount of MDA observed.

Drought-stress-induced in ROS accumulation was accompanied by changes in antioxidant enzyme activity in both the leaves and roots of *C. duriuscula*. However, these changes were rather complex and not the same for various antioxidant enzymes. The only clear trend was in CAT activity (Figure 4A,B), which increased in a dose-dependent manner in both root and leaf tissues. GPX activity showed the same trend but only in roots, not leaves. At the same time, APX activity declined in a dose-dependent manner in both root (Figure 5D) and leaf (Figure 5C) tissues. The SOD activity was minimal under MD and increased under SD treatment (Figure 4E,F), and POD activity increased slightly but did not show any clear dose-dependency (Figure 4C,D). These data are consistent with the concept of high-tissue-specificity of AO enzyme operation [27] and their strong time-dependence. It is also fully in line with the idea that the activity of AO enzymes should be regulated in a manner enabling ROS signaling—e.g., generating ROS waves [8,53] and stress-specific ROS “signatures” [27], to allow adaptation to drought stress (as discussed below in Section 4.4).

### 3.2. The Contribution of Compatible Solutes to Drought Tolerance of C. duriuscula

Increased proline content helps maintain cell turgor and stabilizes the membrane structure under water-deficient conditions [30]. However, some studies have also shown that proline has a limited capacity for osmotic regulation under hyperosmotic stress conditions [54]. In this study, while both MD and SD stress caused an increase in proline content in leaves and roots, its contribution of osmotic adjustment was rather limited. In this context, it could be suggested that proline acted more as a non-enzymatic antioxidant [55] than organic osmolyte. Proline is an effective quencher of ^1^O_2_ production [56]. Proline obtains OH∙ through the H- on its amine group and is further decarboxylated, leading to the formation of pyrrolidin-1-yl [57], thus operating as a non-enzymatic antioxidant. As for the reasons behind this stress-induced increase in proline content, we envisage a mechanism where an increase in H_2_O_2_ content under drought stress induces the biosynthesis of proline. Previous studies have shown that H_2_O_2_ induces the activity of key enzymes Δ1-pyrroline-5-carboxylic acid synthase and glutamate dehydrogenase in the proline synthesis pathway of maize seedlings, and H_2_O_2_ treatment also inhibited the activity of proline dehydrogenase (a key enzyme for proline degradation) [58].

The other major class of compatible solutes is sugars [59]. Drought treatment increased the soluble sugar content in the leaves of soybean [60] and corn [61] and was maintained at a higher level, thus contributing to turgor maintenance [61,62]. Our research found that under MD and SD stress, the soluble sugar content in leaves and roots was higher than that in the control; however, the relative contribution of soluble sugars towards osmotic adjustment declined in both the leaves and roots. In addition to providing plants with energy and metabolic substrates [63], sugars may also operate as a non-enzymatic antioxidants (e.g., via pentose phosphate pathway) to promote ROS scavenging [64]. In addition, sugars are known to be important signal molecules [63,64,65]. Among them, monosaccharides [66,67] and disaccharides [68] play an important role in the response of plants to adverse environmental conditions. Therefore, we suggest that drought-stress induced elevation in sugar content in *C. duriuscula* tissues may be predominantly related to their signalling and ROS scavenging roles, rather than making a major contribution towards osmotic adjustment.

### 3.3. Inorganic Osmolytes Are the Main Players in C. duriuscula Osmotic Adjustment

Under MD stress, the contribution of compatible solutes towards osmotic potential in *C. duriuscula* is decreasing, while the role of inorganic ions becomes more prominent. Osmotic adjustment by means of inorganic osmolytes is considered to be much faster [37] and more energy-efficient [69] compared with de novo synthesis of organic osmolytes. We found that under drought stress, the concentrations of K^+^, Na^+^, and Cl^−^ in *C. duriuscula* leaves and roots were all increased, in a clear, dose-dependent manner. This may increase the cell osmotic potential and reduce water loss. Thus, it appears that *C. duriuscula* adopts the “shortest path” from the rhizosphere environment and apoplasts to absorb K^+^, Na^+^ and Cl^−^ to regulate osmotic pressure, which saves energy and time, making it highly efficient in responding quickly to drought stress.

Of all inorganic ions, K^+^ concentration is highest in plant cells, with free K^+^ concentrations in the cytosol ranging from 100 to 150 mM. Plants also have the capacity to buffer cytosolic K^+^ (for at least several hours [70]), using vacoular reserves. This need for maintenance of K^+^ homeostasis is explained by the significant metabolic roles of this element [71] as well as its role as a second messenger [72]. However, maintaining high cytosolic K^+^ concentrations comes with the caveat of the need for energy-dependent K^+^ uptake under stress conditions [69,73]. Thus, uptake of Na^+^ (whose concentration in the cytosol is much lower, and who cseoncentration in the soil is relatively high) is energetically more favorable. This is indeed the case for *C. duriuscula*, where the relative contribution of Na^+^ in tissue osmotic potential ranged between 16% and 20% in plant roots (Table 1).

### 3.4. How Can Drought Stress Stimulate Inorganic Ion Uptake by Roots?

The classical view of ROS being “foes” has been revised over the last decade by demonstrations that the stress-induced elevation in ROS content is essential for plant signal transduction and adaptation to stress [8]. Additionally, plant cellular membranes harbor a large number of ROS-inducible ion channels [10,74]. A large bulk of them are classified as non-selective cation channels (NSCC). NSCC are Na^+^-permeable [74] and, thus, might represent a pathway for the stress-induced Na^+^ uptake reported here (Figure 8B). Indeed, the typical concentration of Na^+^ in the cytosol under non-saline conditions does not exceed 10 mM [75], while the concentration of Na^+^ in the soil is in a range of a few millimoles. Given that the value of the plasma membrane potential in plant roots under control conditions is in the range of −100–140 mV [76,77], these conditions favor a thermodynamically passive (channel-mediated) Na^+^ uptake. Thus, one can envisage the scenario when a drought-induced increase in ROS content may stimulate the opening of NSCC and lead to the increased uptake and accumulation of Na^+^ reported in this work. Thus, the drought stress-induced increase in ROS level reported here may be causally related to the increased uptake of a “cheap” inorganic osmolyte Na^+^. However, this increase in the ROS content should not exceed a certain threshold that could lead to damage to key structures; hence the need for orchestrated regulation of AO activity.

While the above model works well for Na^+^, it cannot explain the concurrent increase in Cl^−^ uptake and accumulation (Figure 7E,F and Figure 8C). Chloride uptake under conditions of experiments will be thermodynamically active and require secondary-active transport (e.g., operation of H^+^/Cl^−^ exchanger [78]). As this transporter’s operation relies on the existence of H^+^ gradient across both sides of the membrane, it is plausible to suggest that increased Cl^−^ uptake and accumulation under hyperosmotic conditions (Figure 7 and Figure 8) may be related to stress-induced activation of H^+^-ATPase activity. The H^+^-ATPase is a major electrogenic source in plants, and it was shown that osmotic stress causes rapid, significant, and prolonged hyperpolarisation of the plasma membrane potential [37,79,80,81] and stimulates net H^+^ extrusion [37,82]. The central role of plasma membrane H^+^-pump in cell osmotic adjustment is also supported by experiments with specific inhibitors of ATPase activity [83].

The suggested activation of H^+^-ATPase and associated hyperpolarization of the plasma membrane may be also instrumental for the observed increase in K^+^ uptake (Figure 8A). K^+^ uptake in plants is mediated by high- and low-affinity transport systems that rely on the operation of H^+^/K^+^ symporters and K^+^-permeable channels, respectively [69]. Activation of H^+^-ATPase will create both the driving force for a symporter-mediated K^+^ uptake (e.g., via HAK/KUP exchanger) [84], and the consequent membrane hyperpolarization will activate K^+^-permeable inward-rectifying channels (such as AKT in Arabidopsis). Revealing the molecular identity of these transporters in *C. duriuscula* remains a subject of future studies.

## 4. Materials and Methods

### 4.1. Plant Materials, Growth Conditions, and Treatments

*C. duriuscula* was obtained from Lanxi County, Heilongjiang Province, China (126°22′12″ E, 46°12′57″ N). Plants of the same size were transplanted into the soil (4:1 *w/w*) sand and manure mix (Na^+^: 2.30 ± 0.11 mM; Cl^−^: 1.96 ± 0.10 mM); PVC pots with the upper diameter 10 cm, bottom diameter 8 cm, and height 10 cm). Plants were placed in a greenhouse (average temperature of 26/19 °C day/night, humidity 75–85%, light 200 μmol m^−2^ s^−1^) and were irrigated using half-strength Hoagland solution (Na^+^: 10 mM; Cl^−^: 2 × 10^−4^ mM) twice a week. Experimental design implied 10 pots per treatment, with six plants per pot. After 30 d of cultivation, three to six uniform plants about ~15 cm in height were selected from different pots and used for analysis.

Soil field capacity (FC) was kept within a set range by adding water. This included: 75–80% FC = control (C); 40–45% FC = mild drought (MD) stress; 20–25% FC = severe drought (SD) stress. The duration of stress treatment was 14 days. Water loss was estimated by pot weighing. After the drought treatment, some plants from the SD stress group were subjected to re-watering (RW) treatment, maintaining 75–80% FC for 7 d. After treatment, leaves and roots were wrapped separately in aluminum foil, placed immediately in the liquid nitrogen for 5 min, and stored at −80 °C for further analysis.

Three to six independent sample replicates were performed for each treatment of leaves and roots. The SPSS Statistics 22 software (IBM, New York, NY, USA) was used to process all data. The reported data are presented as mean ± SE (standard error). One-way ANOVA is used to analyze the significance of different treatments (Post hoc tests conducted via Tukey’s HSD, at *p* < 0.05 significance level).

### 4.2. Relative Water Content

Fresh leaf and root samples (200 mg each) were immersed in a distilled water in the glass test tubes and kept overnight at 4 °C. The hydrated samples where then extracted and blotted dry using filter paper (Whatman, Maidstone, Kent, UK). The turgid weight (TW) of plant materials was then obtained. Plant samples were then dried in a drying oven at 80 °C for 48 h to obtain dry weight (DW), and relative water content (RWC) was calculated as per [85].

### 4.3. H_2_O_2_ and O_2^−^_ Content

The concentrations of H_2_O_2_ and O_2^−^_ in root and leaf tissues were determined by colorimetry, using a UV3600 device (Shimadzu Corp., Kyoto, Japan). The H_2_O_2_ concentration was assayed according to [86]. Briefly, 0.1 g of plant material was used to extract H_2_O_2_ with cold acetone. The resulting yellow supernatant was collected and its absorbance was measured at 415 nm to calculate the H_2_O_2_ concentration (μ mol g^−1^ FW) (FW, fresh weight) in each sample based on the standard curve.

The O_2^−^_ concentration was assayed according to [87] with minor modifications: 0.1 g of plant material was added to a 1 mL of ice bath homogenate containing 65 mM phosphate buffer (pH 7.8) and then centrifuged at 5000× *g* for 15 min at 4 °C. A total of 1 mL of the resulting supernatant was thoroughly mixed with 0.9 mL of 65 mM phosphate buffer (pH 7.8) and 0.1 mL of 10 mM hydroxylamine; this mixture was incubated at 25 °C for 30 min. A total of 1 mL of 17 mM sulfanilamide and 1 mL of 7 mM α-naphthylamine were then added to the mixture, which was then incubated at 25 °C for 20 min. The absorbance was then measured at 530 nm, and the O_2^−^_ content (n mol g^−1^ FW) was calculated based on the standard curve.

### 4.4. Malondialdehyde Concentration

The MDA concentration was assayed [88] with a slight modification: 0.1 g of plant material was placed into a 2 mL centrifuge tube, and 5% trichloroacetic acid homogenate was added. The mixture was centrifuged at 14,000× *g* for 25 min, and 0.5 mL of the resulting supernatant was added to 1 mL of 20% trichloroacetic acid (containing 0.5% thiobarbituric acid). The solution was immersed in a water bath at 100 °C for 30 min, centrifuged at 10,000× *g* for 10 min, and then 200 μL of the supernatant was aspirated and transferred to a 96-well plate. Its absorbance was then measured at both 532 and 600 nm, and after subtracting the non-specific absorbance at 600 nm, the MDA concentration (nmol g^−1^ FW) was calculated using the extinction coefficient (155 mM^−1^ cm^−1^) based on the following formula

(1) MDA content (nmol/g FW)=[ΔA × V/(ε × d) × 109]/(W × VS/VKE) = 51.6 × ΔA/W,

where VT is the total volume of the reaction system, 4 × 10^−4^ L; ε = molar extinction coefficient of malondialdehyde, 155 × 103 L/mol/cm; d = 96-well plate optical path, 0.5 cm; VS = sample volume, 0.1 mL; VKE = the volume of the extract, 1 mL; W = sample mass, g.

### 4.5. Chlorophyll Extraction and Quantification

The concentrations of chlorophyll *a* and *b* were assayed as described elsewhere [89] using commercial detection kits (CPL-1-G, Suzhou Comin Biotechnology Co., Suzhou, China). Briefly, 0.1 g of plant material was placed into a 15 mL centrifuge tube and 10 mL of 95.5% acetone and absolute alcohol (1:1, v/v) was added. This mixture was stored in the dark for about 2 days, until the sample turned completely white. A total of 200 μL of the extract was taken and put into a 96-well plate. The absorbance at 663 and 645 nm was measured using the microplate spectrophotometer (Epoch^TM^ 2, BioTek, Winooski, VT, USA). Chlorophyll content (in mg/g FW) was calculated as

(2) Chl a = (12.7 × A663 − 2.69 × A645) × VE × D/W/1000,

(3) Chl b =(22.9 × A645 − 4.68 × A663) × VE × D/W/1000,

(4) Total chlorophyll content =(20.21 × A645 + 8.02 × A663)× VE × D/W/1000,

where VE is an extract volume; D is an optical path, 0.5 cm; W is the sample mass, g.

### 4.6. Antioxidant Enzyme Activity

The activities of CAT, SOD, POD, GPX and APX were assayed using detection kits (CAT-1-Y, SOD-1-Y, POD-1-Y, GPX-1-Y and APX-1-Y, respectively; Suzhou Comin Biotechnology Co., Suzhou, China). All the kits have the same extraction solution, but different working solutions. All antioxidant enzyme activities were detected and analyzed according to the protocol and calculation method of these kits. The microplate spectrophotometer (Epoch^TM^ 2, BioTek, Winooski, VT, USA) was used to detect all antioxidant enzyme activities.

### 4.7. Osmolytes Content

Proline concentration (μmol g^−1^ FW) was determined according to [90] with minor modifications. Briefly, 0.1 g of plant material was added to 5 mL sulfosalicylic acid (3% *w*/*v*) homogenate and centrifuged at 4000× *g* for 10 min. A total of 2 mL of the resulting supernatant was placed in a glass test tube, and 2 mL acid-ninhydrin and 2 mL glacial acetic acid were added. The mixture was incubated at 98 °C for 30 min, and cooled to 25 °C. Then, 5 mL of toluene was added to the mixture. The upper layer of the solution containing proline and toluene was transferred to a new centrifuge tube. A total of 0.2 mL of the upper layer solution was placed into a 96-well UV plate. The absorbance at 520 nm wavelength was measured, and the proline content was calculated based on the standard curve.

Soluble sugars concentration (μmol g^−1^ FW) was determined as per [91] with a slight modification: 0.1 g of plant material was added to 5 mL of ice water containing 80% (*v*/*v*) ethanol homogenate for extraction three times; 3 mL of the supernatant was filtered through a 0.45 μm filter paper (Whatman, Maidstone, Kent, UK), the filtrate was recovered and kept at 40 °C drying under a vacuum. The residue was dissolved with distilled water, and the soluble sugars content in the dissolving solution was then determined by high-performance liquid chromatography (HPLC).

The soluble proteins concentration (mg g^−1^ FW) of leaves and roots was determined according to [92] with a slight modification: 0.1 g of plant material was pulverized in liquid nitrogen; the powder was transferred to a centrifuge tube, and 10 mL of SPB (pH 7.0) containing 1 mM EDTA-Na_2_ and 2% (*w*/*v*) polyethylene pyrrolidone was added immediately. The mixture was centrifuged at 11,000× *g* for 15 min at 4 °C. Then, 100 μL of the supernatant was mixed with Bradford solution (Sigma-Aldrich, St. Louis, MO, USA). Then, absorbance was measured at 595 nm and soluble proteins content was calculated using a bovine serum albumin standard curve.

### 4.8. Determination of Ion Concentration and Osmolarity

The sap osmolarity of leaf and root samples was measured as reported elsewhere. Approximately 5 g of each frozen sample were thawed and squeezed to release sap. A total of 10 μL of the sap was then taken and used for osmolarity measurements (in mOsmol kg^−1^) with an osmometer (Vapro 5600, Wescor Inc., Logan, UT, USA). Another 20 μL of the sap sample was diluted and used to measure K^+^ and Na^+^ concentrations (mmol L^−1^ FW) with a flame photometer (AA-6200, Shimadzu, Japan). Chloride concentration was measured with a smart chloride ion meter (CLS-10B, Haiheng Electronic Technology Ltd., Shanghai, China). The relative contribution of organic and inorganic osmolytes towards cell osmotic potential was estimated.

### 4.9. Ion Flux Measurements

Net K^+^, Na^+^, Cl^−^ fluxes across the root surface were measured using the Dynamic Ion Flux Test Equipment (DIFTE) developed by the National Engineering Research Center for Information Technology in Agriculture (NERCITA) in Beijing, China, as described elsewhere [93,94]. A Flexible Dip-type Reference Electrode was used (MI-402: Microelectrodes, Inc., Bedford, NH, USA). For microelectrodes fabrication, the glass capillary tube (BF150-86-10: Sutter Instruments, Novato, CA, USA) was pulled to form a tip of 4–5 μm diameter using a microelectrode puller (P-97: Sutter Instruments, Novato, CA, USA). Pulled capillaries were silanized and then backfilled with appropriate solution (100 mM KCl for K^+^; 250 mM NaCl for Na^+^; 100 mM KCl for Cl^−^). The tip of the microelectrode was then filled with liquid ion exchanger (K^+^, XY-SJ-K; Na^+^, XY-SJ-Na; Cl^−^, XY-SJ-Cl; Xuyue (Beijing) Sci. and Tech. Co., Ltd., Beijing, China). Electrodes were then calibrated in the appropriate set of buffers. Only electrodes with a Nernst slope above 55 mV/decade and correlation r = 0.999 were used. The ion flux is calculated according to the Fick’s first law of diffusion [94].

To detect ion flux (pmol cm^−2^ s^−1^), the root surface was cleaned carefully with distilled water and immersed in the test solution (see below) for about 30 min. Each root to be tested was immobilized using a 1.0 × 1.0 × 0.3 cm epoxy block to prevent it from drifting. Net ion fluxes were then measured from the root meristem (100 μm from the root tips). After recording steady net ion fluxes for 4–5 min, mannitol stock was added to the chamber, to give a final concentration of 40 (MD) and 70 mM (SD), followed by 45–55 min of measurements.

The composition of the test buffers was as follows: 0.1 mM KCl, 0.1 mM CaCl_2_, 0.1 mM MgCl_2_, 0.5 mM NaCl, 0.2 mM Na_2_SO_4_ and 0.3 mM MES, pH 6.0.

## Figures and Tables

**Table 1:** The relative contribution of compatible solutes and inorganic osmolytes towards osmotic adjustment in *C. duriuscula* leaves and roots.

Organs	T	Osmolarity	K^+^ %	Na^+^ %	Cl^−^ %	Ions %	Pro %	SS %	O %	CS %
leaf	C	650 ± 18	30	2	2	34	0.04	21	45	66
	MD	1327 ± 2	21	9	12	42	0.02	12	46	58
	SD	1823 ± 58	16	9	12	39	0.04	12	49	61
	RW	717 ± 29	29	3	3	35	0.04	21	44	65
Root	C	564 ± 7	34	3	3	40	0.06	9	51	60
	MD	1238 ± 15	20	16	21	57	0.04	4	38	43
	SD	1513 ± 18	19	20	25	64	0.04	4	32	36
	RW	568 ± 7.7	35	4	4	43	0.06	8	49	57

Treatments (T): C-control, MD-mild stress, SD-severe stress, RW-re-watering. Ions: overall contribution from inorganic ions (K^+^, Na^+^, Cl^−^). Pro: proline. SS: soluble sugars. O: other organic compounds. CS: overall contribution from compatible solutes. Data are means ± SE (*n* = 3–6).

## Data Availability

The data presented in this study are available on request from the corresponding author.
